# 1851. ID Fellow Quality Improvement Project Shows Gains in Oral Health Exams for People with HIV

**DOI:** 10.1093/ofid/ofad500.1679

**Published:** 2023-11-27

**Authors:** Terrence Park, Lauren Richey, Paula Seal

**Affiliations:** Louisiana State University Health Sciences Center, New Orleans, Louisiana; LSU Health Sciences Center New Orleans, New Orleans, Louisiana; Infectious Diseases Section, LSUHSC, New Orleans, Louisiana

## Abstract

**Background:**

Oral Health is important in people with HIV and remains an important HRSA performance measure. This HRSA measure is the percentage of people with HIV who had an oral health exam by a dentist in the performance year out of the people who had a medical visit in that same year. Our clinic is unique in that it has a Ryan White funded dental clinic with 3 full-time dentists on site. The percentage of patients receiving an oral health exam had decreased in recent years due to shutdowns and fear during the COVID-19 pandemic as well as the vaccine and testing requirements implemented for safety.

**Methods:**

A quality improvement project was designed by an infectious diseases fellow to improve the number of patients receiving an oral health exam. The project was multipronged. The first step was educating the HIV providers about how to place a referral to the HIV dental clinic. The second was placing educational posters in each exam room to educate the patients about the importance of oral health and encourage them to ask for a referral. The last was giving each patient a questionnaire when they checked in for their visit. Each patient filled in the date of their last oral health exam, where it was received, and willingness to be referred for an oral health exam if not examined in the past 12 months. The questionnaires were collected by the provider and served as a reminder to refer. Our goal was a 40% improvement over our baseline, which was 23% in 2020 (goal >32.2%). The intervention began in September of 2022.

**Results:**

In 2019, 31% of our HIV primary care patients were seen in the attached dental clinic, this dropped to 23% in 2020 and 24% in 2021. In the three months prior to our intervention, 38% of patients were referred, and 286 patients had an oral health exam; during the intervention 56%(n=388) of patients were referred, and 329 had an oral health exam. In 2022, 33% of HIV primary care patients were seen in the attached dental clinic, showing an increase despite only three months of the intervention.

**Conclusion:**

Early results indicate an increase in the referrals and an oral health exams with this quality improvement intervention. Outcomes were limited by significant no shows and confounded by removal of COVID vaccine and testing requirements.

**Disclosures:**

**All Authors**: No reported disclosures

